# Characterization of 10,12-pentacosadiynoic acid Langmuir–Blodgett monolayers and their use in metal–insulator–metal tunnel devices

**DOI:** 10.3762/bjnano.5.233

**Published:** 2014-11-26

**Authors:** Saumya Sharma, Mohamad Khawaja, Manoj K Ram, D Yogi Goswami, Elias Stefanakos

**Affiliations:** 1 Clean Energy Research Center, College of Engineering, University of South Florida, Tampa, FL 33620, USA

**Keywords:** Langmuir–Blodgett monolayer, tunnel devices, ultrathin insulator

## Abstract

The characterization of Langmuir–Blodgett thin films of 10,12-pentacosadiynoic acid (PDA) and their use in metal–insulator–metal (MIM) devices were studied. The Langmuir monolayer behavior of the PDA film was studied at the air/water interface using surface tension–area isotherms of polymeric and monomeric PDA. Langmuir–Blodgett (LB, vertical deposition) and Langmuir–Schaefer (LS, horizontal deposition) techniques were used to deposit the PDA film on various substrates (glass, quartz, silicon, and nickel-coated film on glass). The electrochemical, electrical and optical properties of the LB and LS PDA films were studied using cyclic voltammetry, current–voltage characteristics (*I*–*V*), and UV–vis and FTIR spectroscopies. Atomic force microscopy measurements were performed in order to analyze the surface morphology and roughness of the films. A MIM tunnel diode was fabricated using a PDA monolayer assembly as the insulating barrier, which was sandwiched between two nickel layers. The precise control of the thickness of the insulating monolayers proved critical for electron tunneling to take place in the MIM structure. The current–voltage characteristics of the MIM diode revealed tunneling behavior in the fabricated Ni–PDA LB film–Ni structures.

## Introduction

Electronic device fabrication often requires thin film deposition processes which require precise control of the material thickness while maintaining conformity of the layer deposited on a solid substrates. The Langmuir–Blodgett (LB) deposition technique is particularly useful for the transfer of organized monolayers onto a substrate with a fine control of the deposition thickness [1–4]. The technique makes use of the amphiphilic nature of molecules dispersed in water (or another subphase) and later transferred onto a substrate. The production of Langmuir–Schaefer (LS) films involves horizontally dipping the substrate into the liquid subphase, whereas in LB deposition, the substrate is perpendicularly lowered into the liquid subphase.

The use of ω-tricosanoic acid-based LB films in metal–insulator–metal (MIM) diodes with a Ag–LB monolayer–Mg structure was studied by Geddes et al [5]. Mochizuki et al. also explored the use of the LB technique in MIM tunnel diodes by deposition of conductive LB films of bis(ethylenedioxy)tetrathiafulvalene which served as a top electrode [6]. Iwamoto reported on the electrical properties of MIM junctions using polyimide LB films over a range of temperatures [7]. Kaneko et al. reported the use of polydiacetylene thin films in MIM and metal–insulator–semiconductor structures. They measured the thermionic emission through MIM diodes with polydiacetylene LB multilayers [8]. Ram et al. also fabricated MIM structures with polyemeraldine LB films sandwiched between silver and ITO glass plates, which resulted in devices which showed non-linear rectification [9].

In this research, LB films of various fatty acids (with amide or alkyl groups) were used for the fabrication of MIM structures. Initially, a high failure rate was observed with short-circuited MIM devices, which was attributed to pinhole defects or damage to underlying insulating monolayers occurring during the top contact deposition. Pinhole defects can be minimized if individual molecules are bonded together to form a cross-linked structure. The selection of the insulating material is very important: it should be amphiphilic, compatible with the LB technique and polymerizable. Such cross-linking can make the film more compact, thereby reducing the intermolecular distance and making the film more resistant to physical damage during the top contact sputtering. Recently, Langmuir monolayer films of 10,12-pentacosadiynoic acid (PDA) were studied as reported in the literature [8,10–11]. A set of experiments were performed regarding the photo-polymerization of PDA monomers using in situ UV light exposure in the LB trough as well as after deposition of the Langmuir monolayer. This resulted in reduced pinhole defects in the film. In this context, monolayers of PDA were analyzed to check the transfer consistency of PDA onto the substrate for the deposition of LB and LS films. After material and film characterization, these LB layers were characterized for suitability for small signal rectification in MIM tunnel diodes.

The Langmuir monolayer behavior of PDA was studied at the air–water interface to find the ideal surface tension for a close-packed film at the water surface. This was followed by deposition of the film on silicon, indium tin oxide (ITO) glass, quartz and nickel substrates for optical spectroscopy, cyclic voltammetry and electrical measurements [12–15]. The optical and electrochemical properties of the films were investigated using FTIR and cyclic voltammetric studies, respectively. The electrochemical analysis of PDA has proved useful in understanding the chemical activity, such as the oxidation–reduction reactions, as well as their reversibility. Cyclic voltammetry was performed on PDA films deposited onto ITO-coated glass substrates in order to gain qualitative information about the electrochemical processes or for insight into electron transfer rates and the kinetic and thermodynamic behavior of the PDA. Such an analysis can potentially aid in the identification of suitable applications of such materials [16]. The Ni–PDA film–Ni structure was studied in order to understand the tunneling behavior in MIM structures.

## Experimental

### Overview of the deposition of PDA, LS films

**Figure 1:**
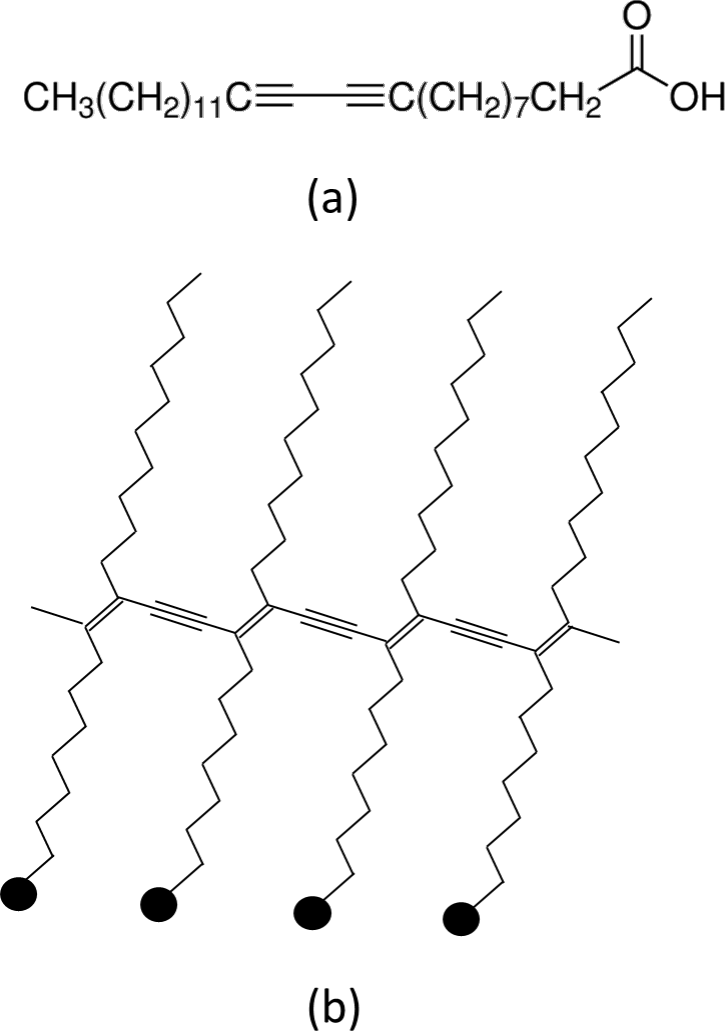
Chemical formula of 10,12-pentacosadiynoic acid is shown in (a). Cross-linking among adjacent monomers is shown in (b).

**Langmuir monolayers and LB films**: For these experiments, PDA was obtained from Sigma-Aldrich in the form of 10,12-pentacosadiynoic acid (≥97.0%, HPLC grade). A 0.2 mg/mL solution of the surfactant PDA was prepared in chloroform (>99.8%, Sigma-Aldrich). Figure 1 shows the molecular structure and UV-polymerized structure of the PDA molecule. The pH value of the water subphase was found to be 6.8. Surface tension–area isotherms were obtained using the KSV NIMA Langmuir–Blodgett Trough system for samples with varying volume and concentration of PDA dissolved in chloroform. The surface tension was measured using a paper Wilhelmy plate suspended in the water in the LB trough. The deposition conditions were regulated using surface tension and compression feedback control. This resulted in the formation of a closely packed film at the air–water interface accomplished by monitoring the change in surface tension with respect to the position of the barriers in the trough. The surface tension–area isotherm of the Langmuir monolayer changes with the variation in the volume of the water-dispersed PDA solution. The Langmuir monolayer formation is dependent on the concentration of the solution and also on the volume of the solution dispersed on the water surface. Several experiments were performed with varying volumes (6, 7, 8 and 10 mL) of a 0.2 mg/mL PDA solution in order to obtain the best monolayer configuration.

**Figure 2:**
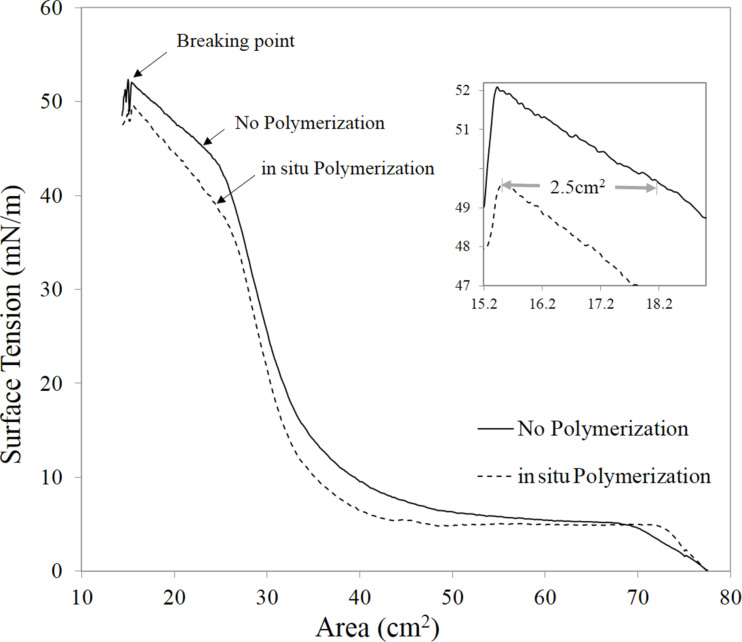
The surface tension–area isotherm of the PDA monolayer with and without in situ polarization in the trough. The inset shows that the in situ polymerization of the PDA film reduces the film surface area by 2.5 cm^2^ (making it 15% more compact), which is close to the maximum confinement before the breaking point.

The quality of the monolayer can be determined by monitoring the surface tension and transfer ratio calculated for each dipping experiment. The transfer ratio, which is defined as the ratio between the decrease in the monolayer area at the water surface during the deposition stage and the area of the substrate, was close to 1 for all experiments. After analysis and repetition of the isotherm as shown in Figure 2, a surface tension of 25 mN/m was selected as the best film configuration for transferring the Langmuir monolayer onto the substrate. The surface tension (and indirectly the film integrity) was efficiently controlled using the KSV NIMA LB Trough controller. For polymerization purposes, the monolayers were exposed to 245 nm UV radiation for 15 min using a UV bench lamp [17].

**Optical characterization**: A Jasco FTIR 4600 was used to perform IR spectroscopy on the PDA film in transmission mode. IR transparent Si was used as a substrate for the purpose of these measurements.

**Electrochemical characterization:** The electrochemical investigation was performed in a cell containing three electrodes. An ITO coated glass substrate with LS/LB films of PDA was used as the working electrode, a platinum wire was used as the counter electrode, and Ag/AgCl as the reference electrode in a 0.01 M HCl electrolyte solution. The electrochemical measurements were made using a Voltalab PGZ301 system.

**MIM diode**: In this experiment, 50 nm of Ni was sputtered onto a silicon wafer with a passivating surface layer of silicon dioxide at a deposition pressure of 3 mTorr. The LB films of 20 and 30 PDA monolayers were deposited onto this Ni-coated substrate using the LB deposition technique [10,18–19]. The Langmuir monolayer was exposed to 254 nm UV radiation to allow cross-linking of the monomers. A confined Ni top contact was sputtered onto the PDA layers with the aid of a shadow mask. To avoid physical damage to the PDA layers, the RF power during sputtering was kept at only 30 W to sustain enough plasma to allow sputtering of Ni atoms. Current–voltage characteristics of the Ni–PDA–Ni assembly were measured using a micromanipulator setup with Dumet (Cu–Fe) probe tips. The 4145B Semiconductor Parameter Analyzer was used to record the *I*–*V* measurements.

## Results and Discussion

In order to analyze the monolayer configuration at the air–water interface of the Langmuir–Blodgett trough, the change in surface tension with respect to film compression was studied. Figure 2 shows the pressure–area isotherm of the PDA Langmuir monolayer with and without in situ polymerization in the trough at the air–water interface. The UV-exposed Langmuir monolayer shows a more compressed area for the same volume of PDA solution dispersed in water. The compactness of the film after in situ polymerization at the surface of the water was improved by 2.5 cm^2^, which is ≈15% of the total surface area of the compressed monolayer. The surface area of the compact film right before the breaking point was recorded as 16.6 cm^2^.

**Figure 3:**
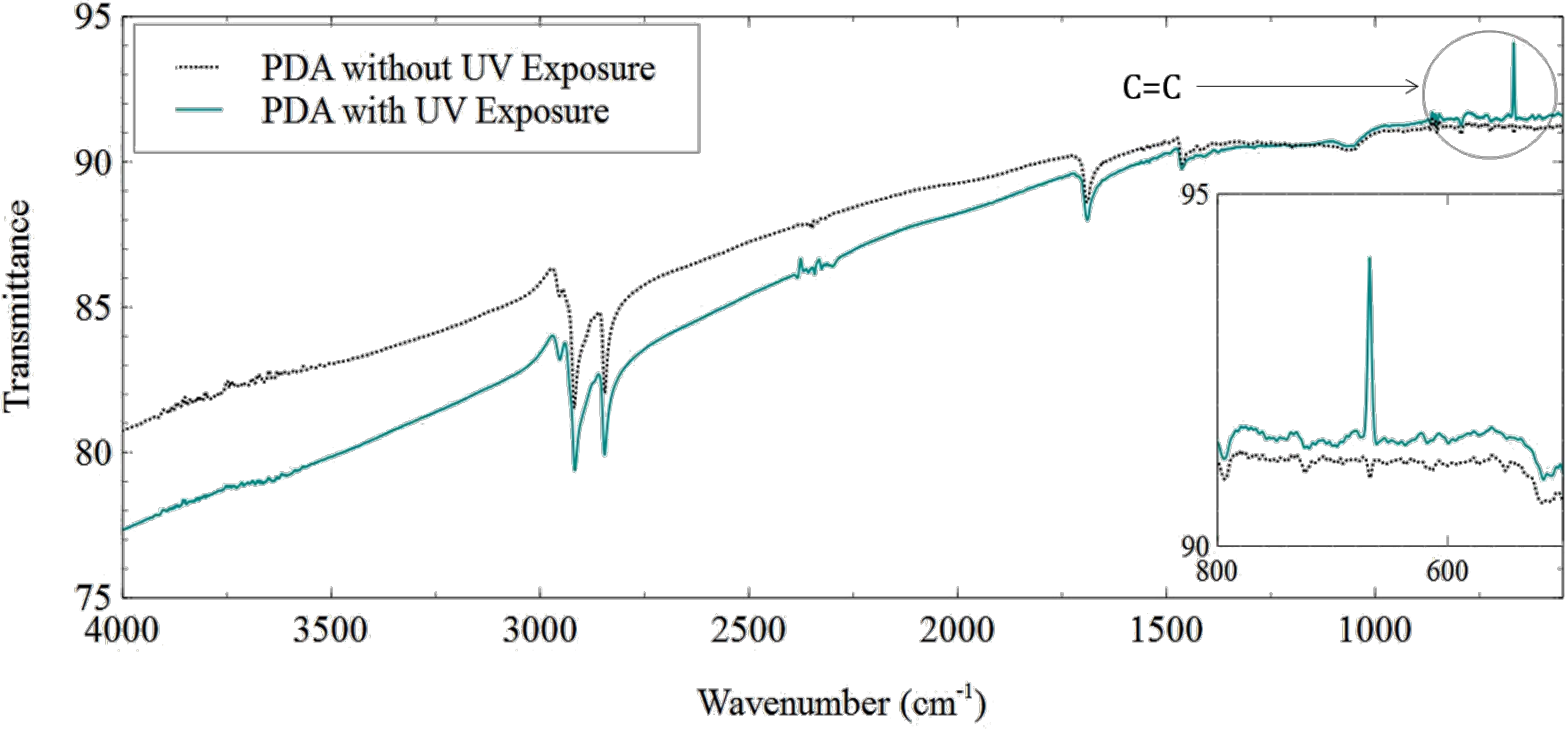
Infrared spectrum of a 20 monolayer PDA sample with and without UV exposure. The inset shows a magnified view of the C=C bending vibrations around 650–750 cm^−1^.

Figure 3 shows the infrared spectra of the 20 monolayer PDA sample. The deposition was performed on an infrared-transparent silicon substrate to characterize the material with respect to its absorption peaks. The analysis was carried out on the basis of the difference between the infrared spectra of a UV-irradiated sample compared to that of the PDA without UV exposure. The peak observed at 1700 cm^−1^ in Figure 3 shows the presence of C=O vibration frequencies. The peaks around 2600–2800 cm^−1^ are attributed to C–H stretching vibrations. There were no C=C peaks observed around ≈700 cm^−1^ in films without UV exposure. However, a distinct C=C peak was seen in the 650–750 cm^−1^ range for UV-irradiated samples, as can be seen in the inset in Figure 3 [20–21].

**Figure 4:**
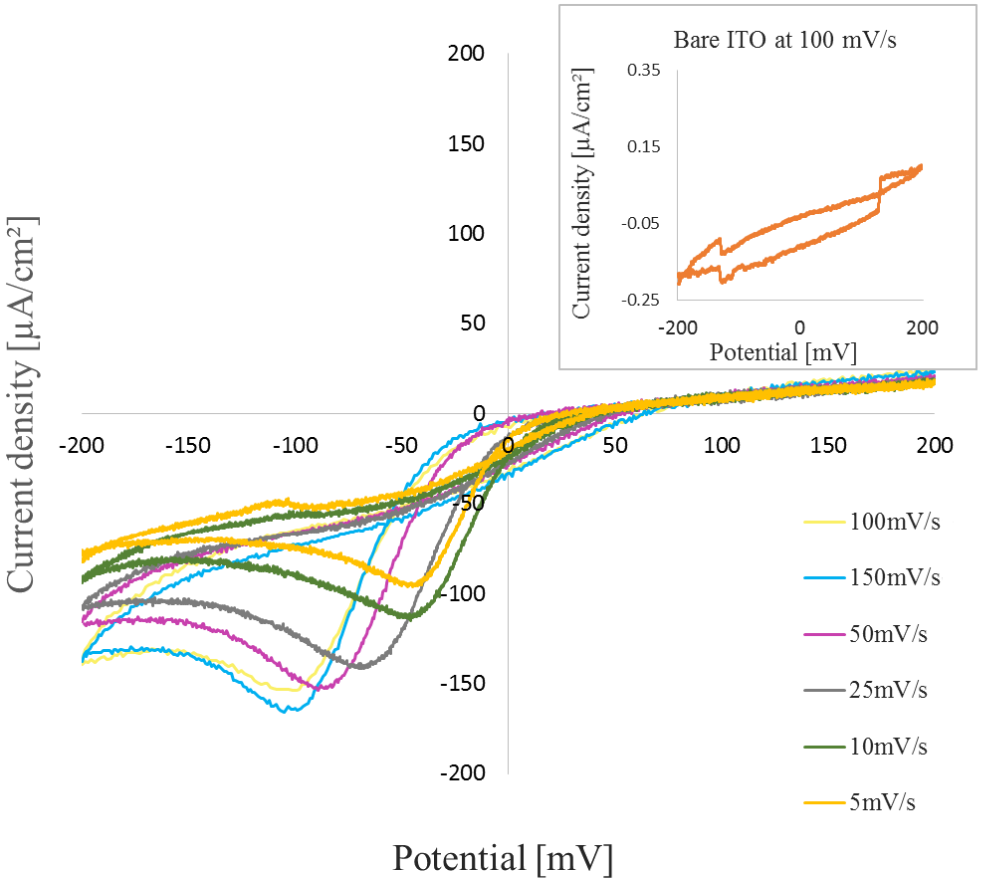
Cyclic voltammetry results with varying scan rates for a 20 monolayer PDA sample in 0.01 M HCl electrolyte. The inset shows the current density vs potential measurements for a bare ITO sample in the same measurement setup.

Figure 4 shows the cyclic voltammogram of the 20 monolayer PDA LB film on ITO coated glass substrates at various scan rates (5, 10, 25, 50, 100, 150 mV/s) in a 0.01 M HCl electrolyte. The PDA shows half-redox characteristics in the CV studies suggesting it is a pseudo-redox material. The redox peak of the PDA LB film shows a distinct shift with an increase in the scan rate from 5 to 150 mV/s. This peak can be attributed to the cross-linking of the vinyl group caused by the UV polymerization during the formation of the Langmuir layer (shown in Figure 1). The vinyl group of the PDA-polymerized molecules exhibits redox properties when interacting with the HCl molecules in the electrolyte. However, even though it is difficult to calculate the diffusion coefficient, the voltammogram is indicative of the presence of the polymerized vinyl group in the Langmuir monolayer. The PDA molecule (10,12-pentacosadiynoic acid) has π bonds which make it electrochemically active in addition to carboxylic acid. The electrolyte interacts with the π bond formation resulting in the pseudo-redox behavior recorded around −150 to 50 mV as a function of scan rate. The redox properties are not intrinsic to the film, but rather, they can be attributed to the reaction between the film and the electrolyte. However, this data clearly indicates that polymerized PDA has electrochemical redox properties. In spite of the π bond conjugation, which provides high carrier mobility, there is still a very low concentration of carriers, as can be seen by the insulating behavior of the material [22–23].

**Figure 5:**
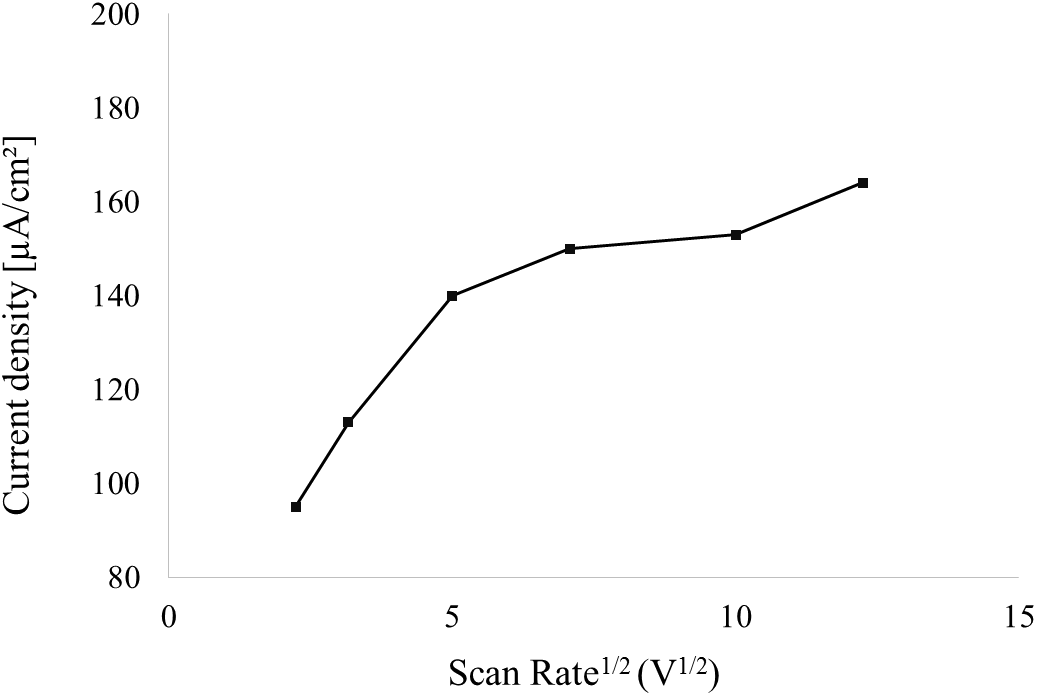
Current density vs voltage scan rate for a 20 monolayer PDA sample on an ITO substrate measured in 0.01 M HCl electrolyte.

Figure 5 shows the relation of the current density to the (scan rate)^1/2^, which was clearly not linear, as would be the case for a redox system.

**Figure 6:**
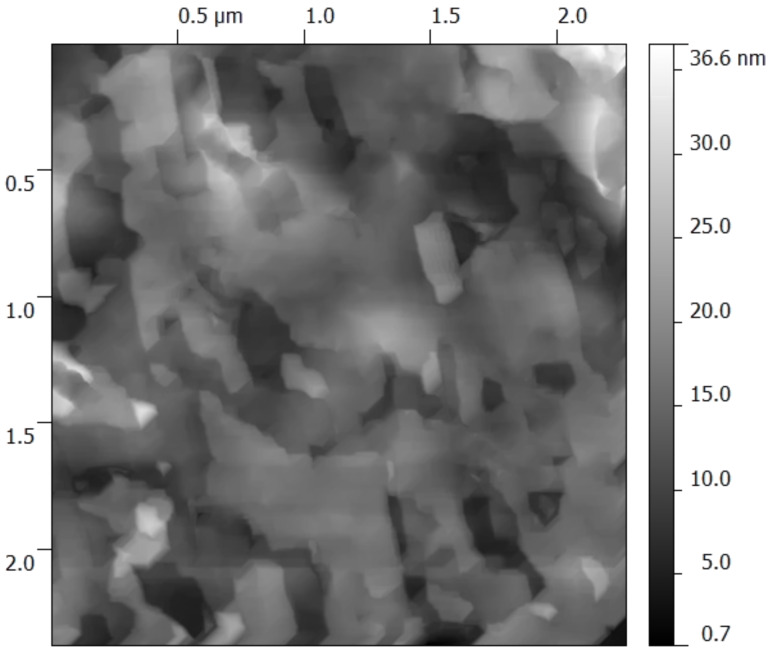
AFM micrograph of 30 monolayer of PDA deposited using the Langmuir–Blodgett technique showing a surface roughness of 34.2 Å. The roughness of the underlying Ni-coated silicon substrate was 18 Å.

The roughness of the deposited monolayers was analyzed using atomic force microscopy (AFM) as shown in Figure 6. Special Bruker AFM tips (0.01–0.025 Ohm·cm, Sb-doped Si) were utilized to scan the film morphology. An average roughness, *R*_a_, of 34.2 Å was measured for 30 layers of PDA. However, this AFM characterization could only provide information about the surface morphology and not the film thickness. The surface roughness of the underlying nickel film was recorded as 18 ± 1 Å [24].

**Figure 7:**
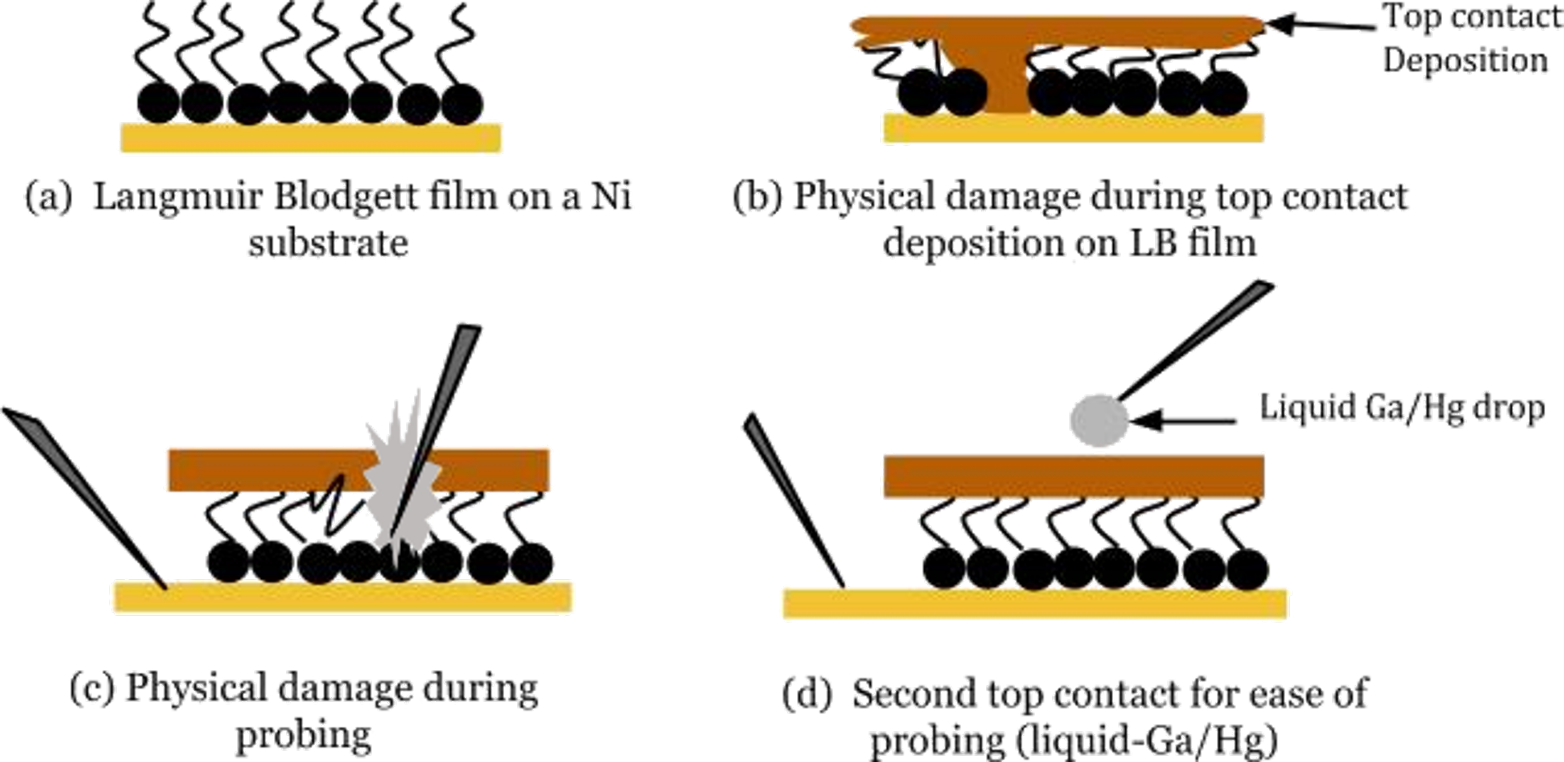
A schematic representation of the challenges in MIM device fabrication using LB monolayers. Typical LB monolayer on a Ni substrate (a); physical damage during top contact deposition (b); probe piercing through underlying metal and insulating layers (c); second liquid-metal contact for easier probing (d).

**Discussion of the MIM structure:** Initially, due to the extreme thinness of the PDA monolayers, it was difficult to avoid pinholes, and most MIM devices failed for this reason. After polymerization of 30 monolayers of PDA Langmuir–Blodgett films, seven out of eight fabricated diodes failed. Figure 7 shows possible fabrication challenges during the production of insulating LB films for MIM devices. A major fabrication challenge was successfully overcome by reducing the RF power during sputtering to as low as 30 W. Higher RF power settings during sputtering of the top contact can potentially cause physical damage to the thin PDA film assembly, resulting in short-circuited MIM devices. Reduction of the RF power during the Ni top contact sputter runs greatly lowered the rate of failed devices, causing less physical damage and avoiding any significant field effect on the PDA film.

Probing on a delicate thin film device was another major challenge. A liquid-metal drop directly on the polymeric film did not allow sufficient repeatability of *I*–*V* measurements. Such a mercury–polymer contact can cause an accumulation of charge on the surface of the mercury drop, thereby causing a change in the effective potential at the interface [4]. To avoid this, Ni contact pads were sputtered to allow probe tip positioning outside of the active area of the device without the need for a liquid top contact. After such optimization, the *I*–*V* characteristics of the Ni–PDA–Ni MIM configuration could be successfully measured using the 4145B Semiconductor Parameter Analyzer and micromanipulator setup to control the probe tips.

The rectification ratio, *RR*, calculated at a bias voltage of 200 mV was as high as ≈110 at ±200 mV. This was calculated as a ratio of currents for an equal voltage deviation around the bias voltage (*V*_b_) as *RR* = *I*_f_ (at *V*_b_ +200 mV)/*I*_r_ (at *V*_b_ −200 mV).

**Figure 8:**
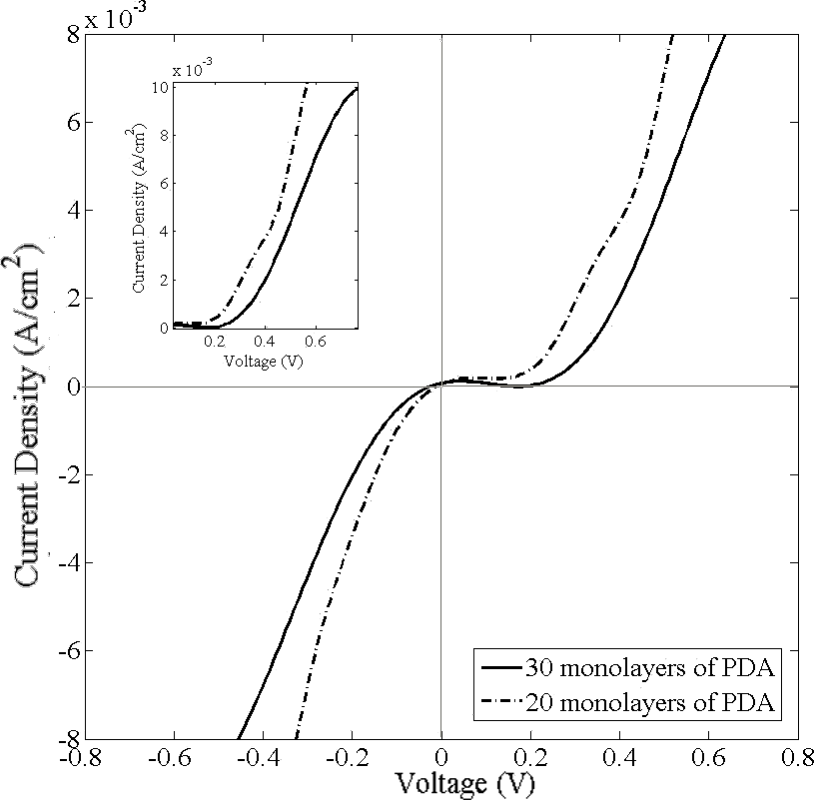
Current density–voltage characteristics for Ni–20 and 30 PDA monolayers–Ni junctions.

Figure 8 shows the *I*–*V* characteristics for 20 to 30 monolayers of PDA in a Ni–PDA–Ni tunnel junction configuration, with an earlier turn-on voltage for 20 monolayer of PDA compared to that for 30 monolayer of PDA.

## Conclusion

The Langmuir–Blodgett film deposition technique was successfully used to deposit highly conformal and less defect-prone PDA monomolecular layers on a solid substrate. A comparison between a UV cross-linked PDA monolayer and one without UV exposure was carried out with the help of infrared spectroscopy, cyclic voltammogram and AFM imaging. An insulating layer of PDA deposited using this technique was used as the thin, insulating layer in a metal–insulator–metal tunnel junction. The top contact deposition and probing procedures were optimized for MIM diode measurements to reduce damage on the underlying PDA monolayer assembly. UV-induced polymerization of PDA introduced intermolecular cross-linking in this layer. A confined Ni top contact was sputtered through a shadow mask at low RF power over the underlying Langmuir–Blodgett film assembly and the *I*–*V* characteristics of the MIM diode were successfully measured. A rectification ratio of ≈110 at ±200 mV was obtained for a bias voltage of 200 mV.
